# Numerical Analysis of Consensus Measures within Groups

**DOI:** 10.3390/e20060408

**Published:** 2018-05-25

**Authors:** Jun-Lin Lin

**Affiliations:** 1 Department of Information Management, Yuan Ze University, Taoyuan 32003, Taiwan; jun@saturn.yzu.edu.tw; Tel.: +886-3-463-8800 (ext. 2611); 2 Innovation Center for Big Data and Digital Convergence, Yuan Ze University, Taoyuan 32003, Taiwan

**Keywords:** consensus measure, Likert scale, variance

## Abstract

Measuring the consensus for a group of ordinal-type responses is of practical importance in decision making. Many consensus measures appear in the literature, but they sometimes provide inconsistent results. Therefore, it is crucial to compare these consensus measures, and analyze their relationships. In this study, we targeted five consensus measures: $\Phi_{e}$ (from entropy), $\Phi_{1}$ (from absolute deviation), $\Phi_{2}$ (from variance), $\Phi_{3}$ (from skewness), and $\Phi_{mv}$ (from conditional probability). We generated 316,251 probability distributions, and analyzed the relationships among their consensus values. Our results showed that $\Phi_{1},{\;\Phi }_{e},{\;\Phi }_{2},$ and $\Phi_{3}$ tended to provide consistent results, and the ordering $\Phi_{1}\leq \Phi_{e}\leq \Phi_{2}\leq \Phi_{3}$ held at a high probability. Although $\Phi_{mv}$ had a positive correlation with $\Phi_{1},{\;\Phi }_{e},{\;\Phi }_{2},$ and $\Phi_{3}$, it had a much lower tolerance for even a small proportion of extreme opposite opinions than $\Phi_{1},{\;\Phi }_{e},{\;\Phi }_{2},$ and $\Phi_{3}$ did.

## 1. Introduction

A consensus measure quantifies the consensus in ratings of a target. It provides fundamental implications of the group’s decision. For example, it can reveal whether the opinions of the group’s members are converging during a successive voting process [1], or whether averaging the members’ ratings to the group level is appropriate [2]. Because of its practicality, the problem of measuring consensus has received much attention, both in academic and applied research [3].

Many consensus measures appear in the literature. Most of them are derived from the deviation of individual ratings from the mean [3,4], while some are based on the extension of entropy [1], or the application of conditional probability [5]. Because consensus measures intend to quantify consensus, one tends to assume that similar conclusions can be drawn using different consensus measures. Although this assumption usually holds, it is still possible that a set of ratings which receives the lowest consensus score using one consensus measure may get a very high consensus score using another consensus measure (see Table 9). It is reasonable that using different consensus measures might lead to different conclusions because they are built on different theoretical concepts. For example, let *A*_1_ and *A*_2_ denote two sets of ratings collected at time t_1_ and t_2_, t_1_ < t_2_. Using one consensus measure might conclude that the consensus of *A*_1_ is smaller than that of *A*_2_ (i.e., the group members’ opinions are converging), but using another consensus measure might yield the opposite conclusion. Therefore, it is crucial to compare these consensus measures in more detail so that one can adequately interpret the meanings of the consensus values.

The objective of this study was to analyze the relationships among different consensus measures so that one can adequately utilize these consensus measures going forward. We first reviewed five consensus measures, and their properties. Then, we took a numerical analysis approach to comparing these consensus measures. This approach proceeded by generating a large number of possible rating distributions, and calculating their consensus scores using each consensus measure. Then, these consensus scores were analyzed to reveal the relationships among these consensus measures. Finally, we discussed how to interpret these consensus scores, and how to select a suitable consensus measure.

## 2. Review of Consensus Measures

### 2.1. Basic Properties of a Consensus Measure

In this paper, we assumed that a rating was an integer in X={1, 2, …,n}. For Likert-type scale responses, *n* = 5 or 7 is often used. Then, the ratings of all group members can be described as a probability distribution p(x) over *X*. Let $p_{i}$ denote the probability p(x=i) of getting a rating *i*. Then,

(1) $$p_{i}\geq 0,\;\mathrm{for}\;i\;=\;1\;\mathrm{to}\;n,$$

(2) $$\sum_{i=1}^{n}p_{i}=1,$$

(3) $$\mathrm{mean}\;m\left(p\right)=\sum_{i=1}^{n}ip_{i},$$

(4) $$\mathrm{variance}\;V\left(p\right)=\sum_{i=1}^{n}p_{i}{\left(i-m\left(p\right)\right)}^{2}.$$

Notably, the rating data are ordinal, and thus, calculating the mean or variance of p(x) is inappropriate. However, mean, variance, or a combination of both was used intensively in the literature to design consensus measures for ordinal attributes.

Let Φ denote a consensus measure, and Φ(p). denote the consensus score of p(x), based on Φ. It is common to restrict the range of Φ(p) between zero and one. This restriction also facilitates comparing different consensus measures. Thus, 0≤Φ(p)≤1, and Φ(p)=1 and Φ(p)=0 indicate the maximum and minimum consensus scores, respectively [5]. In this paper, we divided the consensus measures into three categories, as described in the three subsections below.

### 2.2. Deviation-Based Consensus Measures

Deviation-based consensus measures use the absolute deviation of individual ratings from their mean to measure the consensus. They mainly differ in the power of the absolute deviation. In the literature, power = 1 or 2 was used to measure consensus. In this study, we extended the power to 3.

The average deviation (AD) [6] is the average difference between each rating and the mean, as shown in Equation (5). It is a measure of variability, and its range is between 0 and $\frac{n-1}{2}$, as proven in Corollary 1. Based on AD, we can design a consensus measure $\Phi_{1}\left(p\right)$ such that $0\leq \Phi_{1}\left(p\right)\leq 1$ (see Definition 1).

(5) $$AD\left(p\right)=\;\overset{n}{\underset{i=1}{\sum }}p_{i}\left|i-m\left(p\right)\right|.$$

**Corollary** **1.** *Given a probability distribution* p(x) *over* X={1,2,...,n} *,* $0\leq AD\left(p\right)\leq \frac{n-1}{2}$ *holds.*

**Proof.**  See Appendix A. ☐

**Definition** **1.** *Consensus measure*$\Phi_{1}\left(p\right)=1-\frac{AD\left(p\right)}{\left(n-1\right)/2}$.

Similar to AD, variance (V) is also a measure of variability, and is defined as the average of the squared difference between each rating and the mean, as shown in Equation (4). Its range is between 0 and ${\left(\frac{n-1}{2}\right)}^{2}$, as proven in Corollary 2. Elzinga et al. [4] designed a consensus measure $\Phi_{2}\left(p\right)$ based on V (see Definition 2).

**Corollary** **2.** *Given a probability distribution* p(x) *over* X={1,2,...,n} *,* $0\leq V\left(p\right)\leq {\left(\frac{n-1}{2}\right)}^{2}$ *holds.*

**Proof.**  See Appendix B. ☐

**Definition** **2.** *Consensus measure*$\Phi_{2}\left(p\right)=1-\frac{V\left(p\right)}{{\left(\left(n-1\right)/2\right)}^{2}}$*[4]*.

Notably, AD uses the absolute difference between each rating and the mean, while variance uses the squared difference between each rating and the mean. We can raise the power of the absolute difference to three, and design a new consensus measure $\Phi_{3}\left(p\right)$ as follows: let S denote the average of the cubed absolute difference between each rating and the mean, as shown in Equation (6). The range of S is between 0 and ${\left(\frac{n-1}{2}\right)}^{3}$, as proven in Corollary 3. A consensus measure $\Phi_{3}\left(p\right)$ based on S is shown in Definition 3.

(6) $$S\left(p\right)=\;\sum_{i=1}^{n}p_{i}{\left|i-m\right|}^{3}.$$

**Corollary** **3.** *Given a probability distribution* p(x) *over* X={1, 2,  …,  n} *,* $0\leq S\left(p\right)\leq {\left(\frac{n-1}{2}\right)}^{3}$ *holds.*

**Proof.**  See Appendix C. ☐

**Definition** **3.** *Consensus measure*$\Phi_{3}\left(p\right)=1-\frac{S\left(p\right)}{{\left(\left(n-1\right)/2\right)}^{3}}$.

The maximum values of $\Phi_{1}\left(p\right)$, $\Phi_{2}\left(p\right)$, and $\Phi_{3}\left(p\right)$ all occur when $p_{k}=1$ for some k∈X and $p_{i\in X\backslash \left\{k\right\}}=0$. The minimum values of $\Phi_{1}\left(p\right)$, $\Phi_{2}\left(p\right)$, and $\Phi_{3}\left(p\right)$ all occur when $p_{1}=p_{n}=0.5$, and $p_{i\in X\backslash \left\{1,n\right\}}=0$. Please see the proofs of Corollaries 1, 2, and 3 in Appendix A, Appendix B, and Appendix C, respectively, for details.

Essentially, in $\Phi_{1}\left(p\right)$, $\Phi_{2}\left(p\right)$, and $\Phi_{3}\left(p\right)$, raising the power of the absolute deviation increases the impact of those ratings further from the mean. An example is given below.

**Example** **1.** *Given a probability distribution*p(x)*over*X={1, 2, 3, 4, 5}*where*$p_{i\in X}=0.2$*, a (less consensus) probability distribution*q(x)*with more probabilities further from the mean is generated from*p(x)*by shifting 0.05 probability at*x=4*to*x=5*, i.e.,*$q_{1}=q_{2}=q_{3}=0.2$*,*$q_{4}=0.15$*, and*$q_{5}=0.25$*. Table 1 shows*AD, V, S*,*$\Phi_{1}$*,*$\Phi_{2}$*, and*$\Phi_{3}$*of*p(x)*and*q(x)*. The last row of Table 1 indicates that from*p*to*q*, the consensus is reduced by 0.03 with*$\Phi_{1}$*, 0.03688 with*$\Phi_{2}$*, and 0.04211 with*$\Phi_{3}$. *That is, the impact of increasing the probability further from the mean is greatest in*$\Phi_{3}$*, less in*$\Phi_{2}$*, and least in*$\Phi_{1}$.

### 2.3. Conditional-Probability-Based Consensus Measure

**Figure 1:**
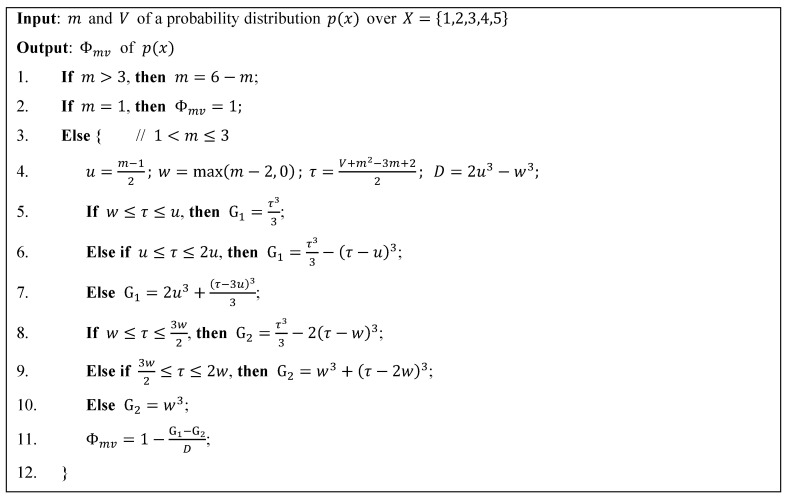
Steps to calculate $\Phi_{mv}\left(p\right)$ for a probability distribution p(x) (revised from Reference [5]).

Corollary 2 shows that the range of variance V is between 0 and ${\left(\frac{n-1}{2}\right)}^{2}$, and the consensus measure $\Phi_{2}$ is constructed based on this range. However, the range of V is a function of the mean m. Specifically, for a given value of m, the range of V is between (m−⎣m⎦)(⎣m⎦+1−m) and (m−1)(n−m), where ⎣m⎦ is the greatest integer ≤n. The size of this range is small as the value of m approaches either end of the interval [1,n], and is large as the value of m approaches the center of the interval [1,n]. Thus, Akiyama et al. [5] proposed a new consensus measure via the conditional probability p(V|m). Because this consensus measure is calculated using both m and V, we denoted it as $\Phi_{mv}\left(p\right)$ in this paper. Figure 1 shows the steps to calculate $\Phi_{mv}\left(p\right)$ for a probability distribution p(x) over X={1, 2, 3, 4, 5}.

Table 2 shows some examples of the probability distribution p(x) with $\Phi_{mv}\left(p\right)=1$ or 0. Unlike $\Phi_{1}$, $\Phi_{2}$, and $\Phi_{3}$, $\Phi_{mv}\left(p\right)=1$ not only occurs when $p_{k}=1$ for some k∈X, and $p_{i\in X\backslash \left\{k\right\}}=0$, but also occurs in many other cases. The first four examples in Table 2 show that the maximum value of $\Phi_{mv}\left(p\right)$ occurs when all probabilities are distributed on one side, and none on the other side of x. Similarly, $\Phi_{mv}\left(p\right)=0$ not only happens when $p_{1}=p_{n}=0.5$, and $p_{i\in X\backslash \left\{1,n\right\}}=0$, but also occurs in many other cases. The last three examples in Table 2 show that a small proportion of extreme opposite opinions can drag $\Phi_{mv}\left(p\right)$ to zero.

### 2.4. Entropy-Based Consensus Measure

In the literature, the Shannon entropy equation and its extensions were used to quantify the diversity of a probability distribution [7]. Given a probability distribution p(x), the Shannon entropy of p(x) is $-\sum_{i=1}^{n}p_{i}\ln\left(p_{i}\right)$ where n is the number of possible values of x, and $p_{i}$ denotes the probability of x=i. Because diversity appears to be the opposite concept of consensus, and the range of the Shannon entropy is between 0 and ln(n), a consensus measure between 0 and 1 based on the Shannon entropy equation can be defined as follows [1,8]:

(7) $$\Phi =1+\frac{\sum_{i=1}^{n}p_{i}\ln\left(p_{i}\right)}{\ln\left(n\right)}.$$

Notably, the Shannon entropy equation treats the variable x as a nominal variable, and not as an ordinal variable; thus, the Shannon entropy equation and Equation (7) are inappropriate for quantifying the consensus of ordinal data, such as Likert-type scale responses. To resolve this problem, Tastle and Wierman [1,8] extended the Shannon entropy equation to define a new consensus measure, denoted as $\Phi_{e}$ in this paper, as follows:

(8) $$\Phi_{e}=1+\sum_{i=1}^{n}p_{i}\log_{2}\left(1-\frac{\left|i-m\right|}{n-1}\right),$$

where m is the mean of p(x), as defined in Equation (3). Similar to $\Phi_{1}\left(p\right)$, $\Phi_{2}\left(p\right)$, and $\Phi_{3}\left(p\right)$, the maximum value of $\Phi_{e}\left(p\right)$ only occurs when $p_{k}=1$ for some k∈X, and $p_{i\in X\backslash \left\{k\right\}}=0$; the minimum value of $\Phi_{e}\left(p\right)$ only occurs when $p_{1}=p_{n}=0.5$, and $p_{i\in X\backslash \left\{1,n\right\}}=0$.

## 3. Experimental Study

### 3.1. Experiment Setup

Given a probability distribution, the five consensus measures reviewed in Section 2 often yielded different consensus scores, and sometimes the differences among these scores were substantial, and led to opposite conclusions. This phenomenon makes it difficult to interpret the meaning of these scores. In this study, we performed a numerical experiment to analyze the relationships among these five consensus measures.

This experiment used the probability distribution p(x) over X={1, 2, 3, 4, 5}, which is common for Likert-type scale data. Specifically, we wrote a small computer program containing a five-level for loop to generate 316,251 probability distributions, where the *i*-th level of the for loop changed the value of $p_{i}$ from 0 to 1 with a step size of 0.2, and cases not satisfying $\sum_{i=1}^{5}p_{i}=1$ were skipped. Thus, these 316,251 probability distributions covered all of the possible probability distributions of p(x) satisfying $p_{i}\in \left\{0,\;0.2,\;0.4,\ldots ,\;0.98,\;1\right\}$ for i = 1 to 5, and $\sum_{i=1}^{5}p_{i}=1$. Then, the consensus scores of each generated probability distribution were calculated and compared to study the relationships among the five consensus measures. Table 3 shows the distribution of the mean values of the 316,251 probability distributions. Most of the generated probability distributions had mean values between 2 and 4.

### 3.2. Correlation

Table 4 shows the Kendall rank correlation coefficients between any two consensus measures. As expected, the results reflected higher than 0.887 correlation between any two consensus measures. That is, if a probability distribution A is ranked higher than another probability distribution B based on one consensus measure, it is very likely that A is also ranked higher than B based on another consensus measure. Let $\tau \left(\Phi_{i},{\;\Phi }_{j}\right)$ denote the Kendall rank correlation coefficient between $\Phi_{i}$ and $\Phi_{j}$. According to Table 4, the lowest correlation occurred at $\tau \left(\Phi_{1},{\;\Phi }_{3}\right)$, and the highest occurs at $\tau \left(\Phi_{1},{\;\Phi }_{e}\right)$. Specifically, $\tau \left(\Phi_{1},{\;\Phi }_{e}\right)>\tau \left(\Phi_{2},{\;\Phi }_{e}\right)>\tau \left(\Phi_{3},{\;\Phi }_{mv}\right)>\tau \left(\Phi_{2},{\;\Phi }_{mv}\right)>\tau \left(\Phi_{2},{\;\Phi }_{3}\right)>\tau \left(\Phi_{1},{\;\Phi }_{2}\right)>\tau \left(\Phi_{e},{\;\Phi }_{mv}\right)>\tau \left(\Phi_{e},{\;\Phi }_{3}\right)>\tau \left(\Phi_{1},{\;\Phi }_{mv}\right)>\tau \left(\Phi_{1},{\;\Phi }_{3}\right)$.

According to Table 3, only 5.18% and 4.82% of the 316,251 generated probability distributions had their mean values in the intervals [1, 2] and (4, 5], respectively. To check whether high correlation still existed for probability distributions with small or large mean values, we calculated the Kendall rank correlation coefficients using both subsets of probability distributions, and the results are shown in Table 5 and Table 6. Every value in Table 5 and Table 6 was smaller than its corresponding value in Table 4. Particularly, $\tau \left(\Phi_{1},{\;\Phi }_{3}\right)$ dropped from 0.887252 in Table 4 to 0.774093 in Table 5, and 0.772132 in Table 6; $\tau \left(\Phi_{1},{\;\Phi }_{mv}\right)$ dropped from 0.925708 in Table 4 to 0.785614 in Table 5, and 0.776873 in Table 6.

### 3.3. Range of Difference

Although Table 4 shows that a positive correlation existed between any two consensus measures of the 316,251 generated probability distributions, some of the generated probability distributions did not follow this general trend. In this section, we calculated the range of differences between two consensus measures to show that this difference was usually small, but was sometimes very big.

Table 7 shows the mean differences between any two consensus measures of the 316,251 generated probability distributions. All of the mean differences were small (<0.167), where the largest mean difference occurred between $\Phi_{1}$ and $\Phi_{3}$, and the smallest mean difference occurred between $\Phi_{1}$ and $\Phi_{e}$. The results were consistent with Table 4, where the smallest and the largest correlation coefficients were $R\left(\Phi_{1},{\;\Phi }_{3}\right)$ and $R\left(\Phi_{1},{\;\Phi }_{e}\right)$, respectively.

Table 8 shows the maximum difference between any two consensus measures of the 316,251 generated probability distributions. Some of the maximum differences were very large. For example, the maximum difference between $\Phi_{mv}$ and other consensus measures was larger than 0.84. Notably, all of the correlation coefficients between $\Phi_{mv}$ and the other consensus measures were greater than 0.92 (see Table 4), and the mean difference between $\Phi_{mv}$ and the other consensus measures was less than 0.16 (see Table 7). Thus, it is reasonable to infer that, although for most probability distributions, the difference between $\Phi_{mv}$ and the other consensus measures was not large, but for some probability distributions, this difference could be huge. Therefore, it is important to understand for which kinds of probability distributions does such a big difference between various consensus measures occur.

The first four examples in Table 9 show some of the generated probability distributions where the maximum differences between two consensus measures occurred. Example 1 had a large proportion (98%) of probability at x=1, thus rendering high consensus scores using $\Phi_{1},{\;\Phi }_{e},\Phi_{2}$, and $\Phi_{3}$. However, this large proportion of probability at x=1 also made values of m close to 1, where m was the mean of the probability distribution. As discussed in Section 2.3, the range of variance is small when m approaches either end of the interval [0, 1]. Thus, for values of m close to 1, the range of variance was small, making $\Phi_{mv}$ very sensitive to even a small proportion of probability at the opposite end of x (2% at x=5 in this example). As a result, Example 1 yielded $\Phi_{mv}=0$. This example was also one of the probability distributions among the 316,251 generated probability distributions that had the maximum difference (in Table 8) between $\Phi_{mv}$ and other consensus measures.

Examples 2 and 3 in Table 9 were similar to Example 1, where a large proportion of probability occurred at x=1, and a small proportion of probability occurred at x=5. The values of $\Phi_{mv}$ remained 0 for Examples 2 and 3. However, the difference between $p_{1}$ and $p_{5}$ decreased from Example 1 through to Example 3, making $\Phi_{1},{\;\Phi }_{e},\Phi_{2}$, and $\Phi_{3}$ smaller for Examples 2 and 3 than for Example 1. Notably, Example 2 was one of the probability distributions that had the maximum difference (in Table 8) between $\Phi_{1}$ and $\Phi_{e}$; Example 3 was one of the probability distributions that had the maximum difference between $\Phi_{2}$ and $\Phi_{3}$.

Example 4 had $p_{3}=p_{5}=0.5$, and yielded the maximum difference (in Table 8) between $\Phi_{1}$ and $\Phi_{2}$, between $\Phi_{1}$ and $\Phi_{3}$, between $\Phi_{e}$ and $\Phi_{2}$, and between $\Phi_{e}$ and $\Phi_{3}$. Suppose that the first four examples in Table 9 describe the voting results at four different stages during a successive voting process. From Example 1 through to Example 4, the value of $\Phi_{1}$ decreased, indicating the group’s consensus was diverging. However, using $\Phi_{mv}$ concluded the opposite. For $\Phi_{e}$, $\Phi_{2}$, and $\Phi_{3}$, the consensus first decreased (from Example 1 through to Example 3), and then increased (from Example 4 onward). However, the differences between the consensus values in Examples 1 and 4 were 0.273596 with $\Phi_{e}$, 0.1716 with $\Phi_{2}$, and −0.02565 with $\Phi_{3}$. Thus, using different consensus measures could lead to different conclusions.

A small change in the probability distribution could result in a different impact on different consensus measures. Consider Examples 1, 7, and 6. They differed by moving a small proportion (2%) of probability from x=5, to x=4, and to x=3, respectively. Although they were similar probability distributions, the value of $\Phi_{mv}$ was 0 in Example 1, and gradually increased to 0.166667 in Example 7, but quickly increased to 0.833333 in Example 6. However, the values of $\Phi_{1},{\;\Phi }_{e},\Phi_{2}$, and $\Phi_{3}$ did not change much among these three examples. Notably, the proportion of probabilities further from the mean had a greater negative impact on $\Phi_{3}$, than on $\Phi_{2}$ and $\Phi_{1}$. Thus, by moving 2% of probability from x=5 to x=4 (i.e., moving closer to the mean), the ordering of $\Phi_{1},{\;\Phi }_{2}$, and $\Phi_{3}$ changed from $\Phi_{3}<\Phi_{2}=\Phi_{1}$ in Example 1 to $\Phi_{3}<\Phi_{1}<\Phi_{2}$ in Example 7. Then, by moving 2% of probability from x=4 to x=5, the ordering of $\Phi_{1},{\;\Phi }_{2}$, and $\Phi_{3}$ changed to $\Phi_{1}<\Phi_{2}<\Phi_{3}$ in Example 6.

The ordering of the values of these consensus measures depended on the probability distribution. For Examples 4, 5, and 6, the value of $\Phi_{mv}$ was the same, but $\Phi_{1}<\Phi_{e}<\Phi_{2}<\Phi_{mv}<\Phi_{3}$ held in Example 4, $\Phi_{e}<\Phi_{1}<\Phi_{mv}<\Phi_{3}<\Phi_{2}$ held in Example 5, and $\Phi_{mv}<\Phi_{1}<\Phi_{e}<\Phi_{2}<\Phi_{3}$ held in Example 6. In Example 7, $\Phi_{mv}$ was the smallest among all consensus measures; however, in Example 8, $\Phi_{mv}$ was the greatest.

### 3.4. Ordering

**Figure 2:**
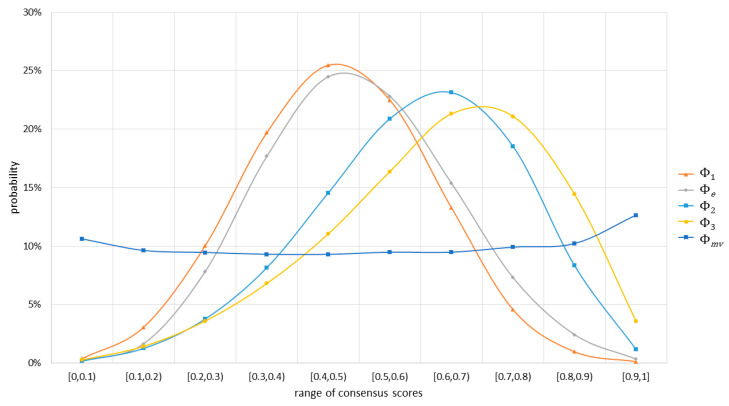
Distributions of consensus scores based on different consensus measures.

From the examples in Table 9, it appeared that no fixed ordering existed among the consensus scores calculated using different consensus measures. Figure 2 shows the distributions of consensus scores of the 316,251 probability distributions generated in this experiment. The distributions of consensus scores based on $\Phi_{1},{\;\Phi }_{e},{\;\Phi }_{2},$ and $\Phi_{3}$ were similar, but were very different from the distribution of consensus scores based on $\Phi_{mv}$. For the consensus values close to 1, the ordering of the probabilities among $\Phi_{1},{\;\Phi }_{e},{\;\Phi }_{2},$ and $\Phi_{3}$ was $\Phi_{1}<\Phi_{e}<\Phi_{2}<\Phi_{3}$, but for the consensus values close to 0, the ordering of the probabilities became $\Phi_{1}\geq \Phi_{e}\geq \Phi_{2}\geq \Phi_{3}$.

In Table 10, we compared the consensus scores of the 316,251 generated probability distributions, and calculated the probabilities of scores based on one consensus measure being less than or equal to scores based on another consensus measure. According to Table 10, $\Phi_{1}\leq \Phi_{2}$ and $\Phi_{e}\leq \Phi_{2}$ always held, while $\Phi_{2}\leq \Phi_{3}$, $\Phi_{e}\leq \Phi_{3}$, $\Phi_{1}\leq \Phi_{3}$, and $\Phi_{1}\leq \Phi_{e}$ also held at very high probabilities. Thus, $\Phi_{1}\leq \Phi_{e}\leq \Phi_{2}\leq \Phi_{3}$ was the most probable ordering among the scores based on these four consensus measures. The orderings between $\Phi_{mv}$, and $\Phi_{1}$ or $\Phi_{e}$ were not apparent, where $\Phi_{1}\leq \Phi_{mv}$ and $\Phi_{e}\leq \Phi_{mv}$ only held at 58.12% and 52.04% probabilities, respectively. Finally, $\Phi_{2}>\Phi_{mv}$ and $\Phi_{3}>\Phi_{mv}$ were likely to occur because $\Phi_{2}\leq \Phi_{mv}$ and $\Phi_{3}\leq \Phi_{mv}$ held at 36.84% and 28.01% probabilities, respectively.

### 3.5. Relationships

To visually inspect the relationships among different consensus measures, we plotted the consensus values of the 316,251 generated probability distributions in two-dimensional (2D) scatter charts.

**Figure 3:**
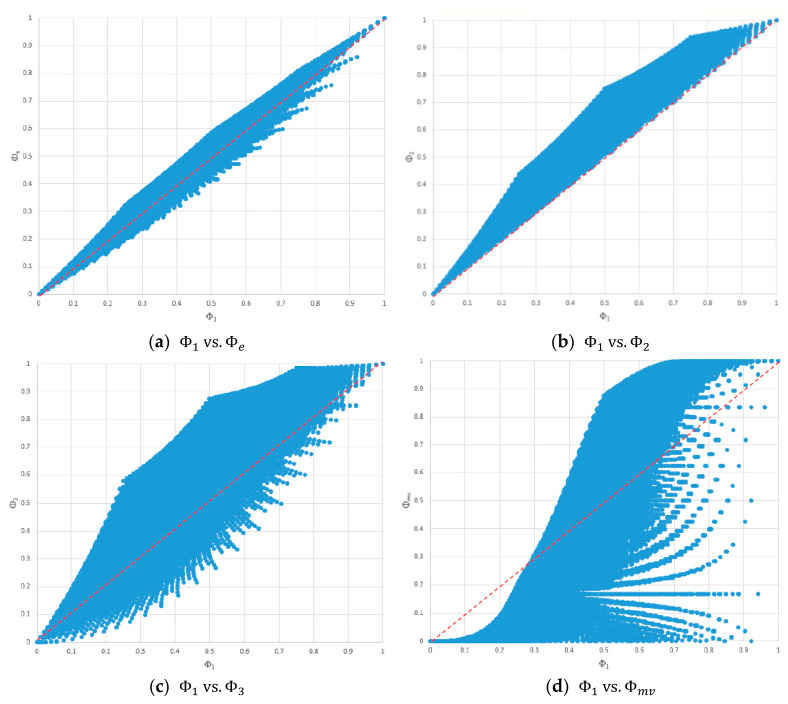
$\Phi_{1}$ vs. other consensus measures. (**a**) $\Phi_{1}\;\mathrm{vs}.{\;\Phi }_{e}$; (**b**) $\Phi_{1}\;\mathrm{vs}.{\;\Phi }_{2}$; (**c**) $\Phi_{1}\;\mathrm{vs}.{\;\Phi }_{3}$; and (**d**) $\Phi_{1}\;\mathrm{vs}.{\;\Phi }_{mv}$.

Figure 3 shows the scatter charts of $\Phi_{1}$ scores versus scores based on the other consensus measures, where the red dashed lines represent equality between two consensus scores. As expected, a positively correlated trend existed. No fixed ordering existed between $\Phi_{1}$ and the other consensus measures except that $\Phi_{1}\leq \Phi_{2}$ always held, as shown in Figure 3b. According to Figure 3a–c, as the value of $\Phi_{1}$ approached 0 or 1, the ranges of $\Phi_{e},{\;\Phi }_{2}$ and $\Phi_{3}$ narrowed, indicating that the maximum differences between $\Phi_{1}$ and $\Phi_{e},{\;\Phi }_{2}$, and $\Phi_{3}$ decreased. However, when the value of $\Phi_{1}$ approached 0.5, the ranges of $\Phi_{e},{\;\Phi }_{2}$, and $\Phi_{3}$ increased, indicating that the maximum differences between $\Phi_{1}$ and $\Phi_{e},{\;\Phi }_{2}$, and $\Phi_{3}$ also increased. Furthermore, the maximum difference between $\Phi_{1}$ and $\Phi_{e}$ was smaller than both the maximum differences between $\Phi_{1}$ and $\Phi_{2}$, and between $\Phi_{1}$ and $\Phi_{3}$.

Figure 3d shows that, for $\Phi_{1}<1$, as the value of $\Phi_{1}$ increased, the range of $\Phi_{mv}$ increased, and the maximum difference between $\Phi_{1}$ and $\Phi_{mv}$ became huge. For any probability distribution satisfying $\Phi_{1}=1$, its $\Phi_{mv}$ was also 1. However, for any probability distribution satisfying $\Phi_{mv}=1$, its value of $\Phi_{1}$ was not necessarily 1. In fact, there were only n probability distributions satisfying $\Phi_{1}=1$, that is, when $p_{k}=1$ for some k∈X, and $p_{i\in X\backslash \left\{k\right\}}=0$ (this statement also applies to $\Phi_{e},\;\Phi_{2}$, and $\Phi_{3}$). However, there were many probability distributions satisfying $\Phi_{mv}=1$ (see Table 2 for examples).

**Figure 4:**
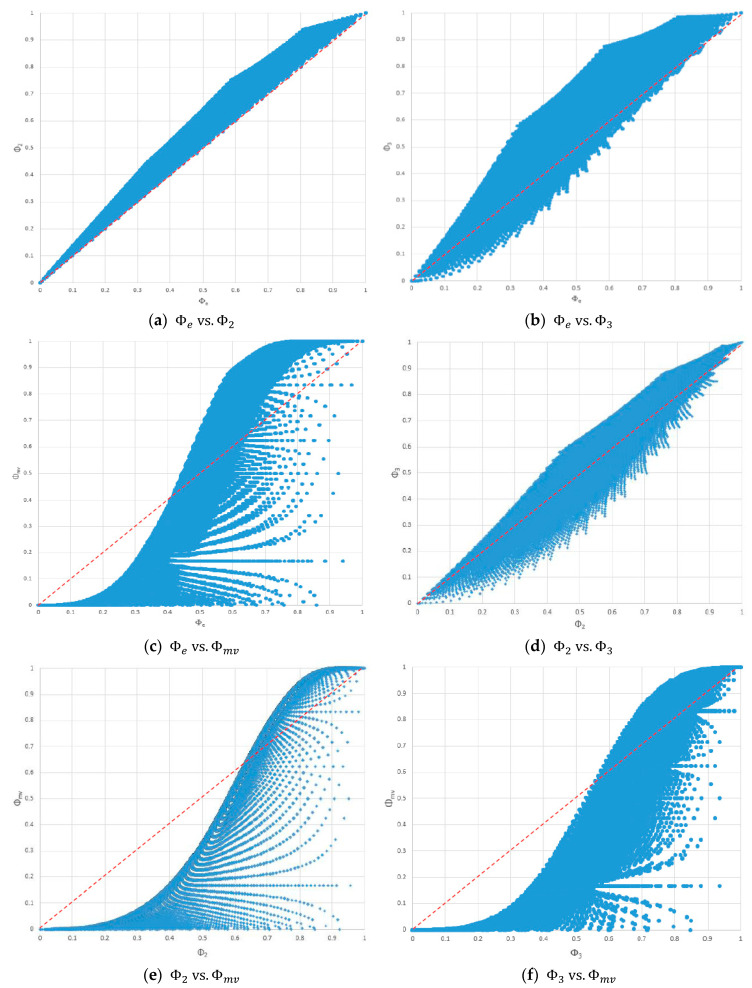
Scatter charts of $\Phi_{e},{\;\Phi }_{2},{\;\Phi }_{3}$, and $\Phi_{mv}$. (**a**) $\Phi_{e}\;\mathrm{vs}.{\;\Phi }_{2}$; (**b**) $\Phi_{e}\;\mathrm{vs}.{\;\Phi }_{3}$; (**c**) $\Phi_{e}\;\mathrm{vs}.{\;\Phi }_{mv}$; (**d**) $\Phi_{2}\;\mathrm{vs}.{\;\Phi }_{3}$; (**e**) $\Phi_{2}\;\mathrm{vs}.{\;\Phi }_{mv}$; and (**f**) $\Phi_{3}\;\mathrm{vs}.{\;\Phi }_{mv}$.

Figure 4 shows the scatter charts of the consensus scores based on $\Phi_{e},{\;\Phi }_{2},{\;\Phi }_{3}$, and $\Phi_{mv}$. No fixed ordering existed among these consensus measures except that $\Phi_{e}\leq \Phi_{2}$ always held, as shown in Figure 4a. According to Figure 4a,b,d, for $\Phi_{e},{\;\Phi }_{2}$, and $\Phi_{3}$, as the value of one consensus measure approached either end of the interval [0, 1], the range of another consensus measure decreased. According to Figure 4a,b, the maximum difference between $\Phi_{e}$ and $\Phi_{2}$ was smaller than that between $\Phi_{e}$ and $\Phi_{3}$. According to Figure 3b and Figure 4a,d, the maximum difference between $\Phi_{2}$ and $\Phi_{e}$ was smaller than those between $\Phi_{2}$ and $\Phi_{1}$, and between $\Phi_{2}$ and $\Phi_{3}$. Figure 4c,e,f show a similar pattern to Figure 3d. As the value of $\Phi_{e}$ (or $\Phi_{2}$, $\Phi_{3}$) increased (before reaching 1), the range of $\Phi_{mv}$ increased, and the maximum difference between $\Phi_{e}$ (or $\Phi_{2}$ and $\Phi_{3}$) and $\Phi_{mv}$ became huge.

## 4. Discussions

Given a probability distribution, using different consensus measures often yields different consensus scores. If there exists a fixed ordering among these scores, then consistent results can be drawn using different consensus measures. Unfortunately, such an ordering depends on the given probability distribution. However, according to Table 10, the following orderings among the consensus scores held at high probabilities: $\Phi_{1}\leq \Phi_{e}\leq \Phi_{2}\leq \Phi_{3}$, $\Phi_{2}>\Phi_{mv}$, and $\Phi_{3}>\Phi_{mv}$.

Because there exists no fixed ordering among consensus scores based on different consensus measures, it is crucial to know the relationships among the consensus measures. Figure 3 and Figure 4 revealed that, for $\Phi_{1},{\;\Phi }_{e},{\;\Phi }_{2},$ and $\Phi_{3}$, as the value of one consensus measure approached either end of the interval [0, 1], the ranges of the other consensus measures decreased. Thus, one can expect smaller differences among $\Phi_{e},\;\Phi_{1},\;\Phi_{2}$, and $\Phi_{3}$ for consensus scores close to 0 or 1, than for consensus scores close to 0.5.

According to Figure 3d and Figure 4c,e,f, the range of $\Phi_{mv}$ increased rapidly as the value of $\Phi_{e},\;\Phi_{1},\;\Phi_{2}$, or $\Phi_{3}$ increased. Thus, $\Phi_{mv}$ often gave results inconsistent with those from $\Phi_{e},\;\Phi_{1},\;\Phi_{2}$, and $\Phi_{3}$, especially when the value of $\Phi_{e},\;\Phi_{1},\;\Phi_{2}$, or $\Phi_{3}$ was large. Looking at these figures from another perspective, the ranges of $\Phi_{1},{\;\Phi }_{e},{\;\Phi }_{2},$ and $\Phi_{3}$ decreased as the value of $\Phi_{mv}$ increased. Notably, $\Phi_{mv}$ tended to give low scores to probability distributions where some probability was located at the opposite end of the mean. Thus, for values of $\Phi_{mv}$ close to zero, one should also check the values of $\Phi_{1},{\;\Phi }_{e},{\;\Phi }_{2},$ and $\Phi_{3}$ for possibly inconsistent results.

Choosing a consensus measure remains a task for the users. If one has a low tolerance for even a small proportion of extreme opposite opinions, then $\Phi_{mv}$ is a good choice. Otherwise, the other consensus measures tend to provide consistent results. If one prefers to emphasize the opinions further from the mean, then $\Phi_{3}$ is a good choice. Otherwise, either $\Phi_{1}$ or $\Phi_{e}$ can be used, both yielding similar results. Finally, $\Phi_{2}$ provides a middle ground between $\Phi_{3}$ and $\Phi_{1}$.

## 5. Conclusions

An understanding of the characteristics of consensus measures helps users interpret results. For example, according to Figure 3b, $\Phi_{1}$ tended to yield a smaller consensus score than $\Phi_{2}$ for the same probability distribution; thus, a probability distribution A with $\Phi_{1}\left(A\right)=0.6$ might have more consensus than another probability distribution B with $\Phi_{2}\left(B\right)=0.7$, even though $\Phi_{1}\left(A\right)<\Phi_{2}\left(B\right)$.

In essence, two opposite forces shape the design of a consensus measure: the force of obeying the majority, and the force of respecting the minority. Consensus measure $\Phi_{e}$ stressed on the former, and the opinion of the minority has a weak impact on the consensus scores. In contrast, $\Phi_{mv}$ emphasizes the latter, and the opinion of the minority substantially influences the consensus scores, as shown in the first four examples in Table 9.

Deviation-based consensus measures (i.e., $\Phi_{1},\;\Phi_{2}$, and $\Phi_{3}$) allow users to adjust the strengths of these two forces. As described in Section 2.2, raising the power of the absolute deviation in the deviation-based consensus measures increases the impact of ratings further from the mean. Intuitively, unless the probabilities of all opinions are distributed evenly on opposite sides of the mean (e.g., $p_{1}$ = $p_{n}$ = 0.5), ratings further from the mean represent the opinions of the minority. Thus, going from $\Phi_{1}$ through to $\Phi_{3}$, the impact of the minority increases. Overall, fine-tuning the balance between the force of obeying the majority, and the force of respecting the minority in a consensus measure provides the consensus measure with more flexibility for various situations, and is a direction of research worth exploring.

## Figures and Tables

**Table 1:** From p(x) to q(x), consensus score reduces the most in $\Phi_{3}$, less in $\Phi_{2}$, and least in $\Phi_{1}$.

	AD	V	S	$\Phi_{1}$	$\Phi_{2}$	$\Phi_{3}$
p(x)	1.2	2	3.6	0.4	0.5	0.55
q(x)	1.26	2.1475	3.9369	0.37	0.463125	0.507888
Φ(p)−Φ(q)	-	-	-	0.03	0.03688	0.04211

**Table 2:** Some examples of the probability distribution p(x) satisfying $\Phi_{mv}\left(p\right)=1$ or 0.

$p_{1}$	$p_{2}$	$p_{3}$	$p_{4}$	$p_{5}$	$\Phi_{mv}\left(p\right)$
1	0	0	0	0	1
0.75	0.25	0	0	0	1
0.50	0.50	0	0	0	1
0	0.96	0.40	0	0	1
0.50	0	0	0	0.50	0
0.90	0	0	0	0.10	0
0.96	0	0	0	0.04	0
0.98	0	0	0	0.02	0

**Table 3:** The distribution of the mean values of the 316,251 generated probability distributions.

Range of Mean	1≤m≤2	2<m≤3	3<m≤4	4<m≤5
Number of probability distributions	16,390	143,747	140,878	15,236
Probability	5.18%	45.45%	44.55%	4.82%

**Table 4:** Kendall rank correlation coefficients between consensus measures using all 316,251 probability distributions.

	$\Phi_{1}$	$\Phi_{e}$	$\Phi_{2}$	$\Phi_{3}$	$\Phi_{mv}$
$\Phi_{1}$	1	0.990202	0.967755	0.887252	0.925708
$\Phi_{e}$	0.990202	1	0.99008	0.940635	0.964478
$\Phi_{2}$	0.967755	0.99008	1	0.969419	0.970876
$\Phi_{3}$	0.887252	0.940635	0.969419	1	0.974605
$\Phi_{mv}$	0.925708	0.964478	0.970876	0.974605	1

**Table 5:** Kendall rank correlation coefficients between consensus measures using the 16,390 probability distributions where 1≤m≤2.

	$\Phi_{1}$	$\Phi_{e}$	$\Phi_{2}$	$\Phi_{3}$	$\Phi_{mv}$
$\Phi_{1}$	1	0.967110	0.930117	0.774093	0.785614
$\Phi_{e}$	0.967110	1	0.985489	0.904147	0.891701
$\Phi_{2}$	0.930117	0.985489	1	0.942186	0.900688
$\Phi_{3}$	0.774093	0.904147	0.942186	1	0.940492
$\Phi_{mv}$	0.785614	0.891701	0.900688	0.940492	1

**Table 6:** Kendall rank correlation coefficients between consensus measures using the 15,236 probability distributions where 4<m≤5.

	$\Phi_{1}$	$\Phi_{e}$	$\Phi_{2}$	$\Phi_{3}$	$\Phi_{mv}$
$\Phi_{1}$	1	0.965686	0.930352	0.772132	0.776873
$\Phi_{e}$	0.965686	1	0.986604	0.905085	0.886878
$\Phi_{2}$	0.930352	0.986604	1	0.941809	0.897223
$\Phi_{3}$	0.772132	0.905085	0.941809	1	0.939574
$\Phi_{mv}$	0.776873	0.886878	0.897223	0.939574	1

**Table 7:** Mean differences between any two consensus measures.

	$\Phi_{1}$	$\Phi_{e}$	$\Phi_{2}$	$\Phi_{3}$	$\Phi_{mv}$
$\Phi_{1}$	0	0.0381011	0.1258246	0.16606489	0.15698299
$\Phi_{e}$	0.0381011	0	0.0895491	0.1281988	0.1429986
$\Phi_{2}$	0.1258246	0.0895491	0	0.0491733	0.14278058
$\Phi_{3}$	0.16606489	0.1281988	0.0491733	0	0.149693
$\Phi_{mv}$	0.15698299	0.1429986	0.14278058	0.149693	0

**Table 8:** Maximum differences between two consensus measures.

	$\Phi_{1}$	$\Phi_{e}$	$\Phi_{2}$	$\Phi_{3}$	$\Phi_{mv}$
$\Phi_{1}$	0	0.108996	0.25	0.375	0.9216
$\Phi_{e}$	0.108996	0	0.165037	0.290037	0.858559
$\Phi_{2}$	0.25	0.165037	0	0.249661	0.9216
$\Phi_{3}$	0.375	0.290037	0.249661	0	0.849347
$\Phi_{mv}$	0.9216	0.858559	0.9216	0.849347	0

**Table 9:** Some examples of the probability distribution p(x), and their consensus scores.

Example Number	$p_{1}$	$p_{2}$	$p_{3}$	$p_{4}$	$p_{5}$	$\Phi_{1}$	$\Phi_{e}$	$\Phi_{2}$	$\Phi_{3}$	$\Phi_{mv}$
1	0.98	0	0	0	0.02	0.9216	0.858559	0.9216	0.849347	0
2	0.90	0	0	0	0.10	0.64	0.531004	0.64	0.4096	0
3	0.86	0	0	0	0.14	0.5184	0.415761	0.5184	0.268739	0
4	0	0	0.50	0	0.50	0.5	0.584963	0.75	0.875	0.833333
5	0.02	0	0	0.16	0.82	0.8032	0.796982	0.8944	0.85691	0.833333
6	0.98	0	0.02	0	0	0.9608	0.966392	0.9804	0.981168	0.833333
7	0.98	0	0	0.02	0	0.9412	0.940313	0.9559	0.936443	0.166667
8	0.	0.96	0	0	0.04	0.8848	0.884354	0.9136	0.880353	0.99176

**Table 10:** The probability of scores based on one consensus measure to be equal to or less than scores based on another consensus measure for the 316,251 generated probability distributions.

≤	$\Phi_{e}$	$\Phi_{2}$	$\Phi_{3}$	$\Phi_{mv}$
$\Phi_{1}$	94.66%	100%	96.41%	58.12%
$\Phi_{e}$	-	100%	96.96%	52.04%
$\Phi_{2}$	-	-	84.35%	36.84%
$\Phi_{3}$	-	-	-	28.01%

## Appendix A

In this section, we derived the range of AD(p), where p is a probability distribution over X={1,2,...,n} with mean m. Lemma 1 shows that, by moving each $p_{i\leq m}$ gradually toward $p_{1}$, the AD of the resulting distribution keeps increasing. Similarly, Lemma 2 shows that by moving each $p_{i>m}$ gradually toward $p_{n}$, the AD of the resulting distribution also keeps increasing.

**Lemma** **1.** *Let*p(x)*and*q(x)*be two probability distributions over*X={1,2,...,n}*,*$p_{i}$*and*$q_{i}$*denote*p(x=i)*and*q(x=i)*, respectively, and*$p_{i}<1$*and*$q_{i}<1$*for each*i∈X*. Let*$m=\sum_{i=1}^{n}ip_{i}$*, and*k*denote the greatest integer satisfying*1<k≤m*and*$p_{k}>0$*. If*$q_{k-1}=p_{k}+p_{k-1}$*,*$q_{k}=0$*, and*$q_{i}=p_{i}$*for each*i∈X\{k−1,k}*, then*AD(q)>AD(p).

**Proof.** By Equation (3), the mean of q(x) is

$$m^{\prime }=\left(\sum_{i=1}^{k-2}ip_{i}\right)+\left(k-1\right)\left(p_{k-1}+p_{k}\right)+\left(\sum_{i=k+1}^{n}ip_{i}\right)=\left(\sum_{i=1}^{n}ip_{i}\right)-p_{k}=m-p_{k}.$$

Let j denote the smallest integer such that m<j and $p_{j}>0$. Then, $p_{i}=0$ for k+1≤i≤j−1, and $q_{i}=0$ for k≤i≤j−1. Thus,

$$\sum_{i=k+1}^{j-1}|i-m|p_{i}=\sum_{i=k}^{j-1}\left|i-m^{\prime }\right|q_{i}=0.$$

Also, $0<p_{k}<1$, k≤m, and m<j yield $k-1\leq m-1<m^{\prime }<m<j$.

$$\begin{array}{l} AD\left(q\right)=\left(\sum_{i=1}^{k-2}(m^{\prime }-i)p_{i}\right)+\left(\left(m^{\prime }-(k-1\right)\right)\left(p_{k-1}+p_{k}\right)+\left(\sum_{i=k}^{j-1}|i-m^{\prime }|q_{i}\right)+\left(\sum_{i=j}^{n}(i-m^{\prime })p_{i}\right)\\ =\left(\sum_{i=1}^{k-2}(m^{\prime }-i)p_{i}\right)+\left(\left(m^{\prime }-(k-1\right)\right)\left(p_{k-1}+p_{k}\right)+\left(\sum_{i=k+1}^{j-1}|i-m|p_{i}\right)+\left(\sum_{i=j}^{n}(i-m^{\prime })p_{i}\right)\\ =\left(\left(\sum_{i=1}^{k-2}(m-i)p_{i}\right)-p_{k}\left(\sum_{i=1}^{k-2}p_{i}\right)\right)+\left(m-p_{k}-\left(k-1\right)\right)\left(p_{k-1}+p_{k}\right)+\left(\sum_{i=k+1}^{j-1}|i-m|p_{i}\right)+\left(\left(\sum_{i=j}^{n}(i-m)p_{i}\right)+p_{k}\left(\sum_{i=j}^{n}p_{i}\right)\right)\\ =\left(\sum_{i=1}^{k-2}(m-i)p_{i}\right)-p_{k}\left(\sum_{i=1}^{k-2}p_{i}\right)+\left(m-\left(k-1\right)\right)p_{k-1}+\left(m-k\right)p_{k}+p_{k}-p_{k}\left(p_{k-1}+p_{k}\right)+\left(\sum_{i=k+1}^{j-1}|i-m|p_{i}\right)+\left(\sum_{i=j}^{n}(i-m)p_{i}\right)+p_{k}\left(\sum_{i=j}^{n}p_{i}\right)\\ =AD\left(p\right)+p_{k}\left(\left(-\sum_{i=1}^{k-2}p_{i}\right)+1-\left(p_{k-1}+p_{k}\right)+\left(\sum_{i=j}^{n}p_{i}\right)\right)\;\\ =AD\left(p\right)+p_{k}\left(1-\sum_{i=1}^{k}p_{i}+\sum_{i=j}^{n}p_{i}\right)=AD\left(p\right)+2p_{k}\sum_{i=j}^{n}p_{i}>AD\left(p\right). \end{array}$$

 ☐

**Lemma** **2.** *Let*p(x)*and*q(x)*be two probability distributions over*X={1,2,...,n}*,*$p_{i}$*and*$q_{i}$*denote*p(x=i)*and*q(x=i)*, respectively, and*$p_{i}<1$*and*$q_{i}<1$*for each*i∈X*. Let*$m=\sum_{i=1}^{n}ip_{i}$*, and*j*denote the smallest integer satisfying*m<j<n*and*$p_{j}>0$*. If*$q_{j}=0$*,*$q_{j+1}=p_{j}+p_{j+1}$*, and*$q_{i}=p_{i}$*for each*i∈X\{j,j+1}*, then*AD(q)>AD(p).

**Proof.** By Equation (3), the mean of q(x) is

$$m^{\prime }=\left(\sum_{i=1}^{j-1}ip_{i}\right)+\left(j+1\right)\left(p_{j}+p_{j+1}\right)+\left(\sum_{i=j+2}^{n}ip_{i}\right)=\left(\sum_{i=1}^{n}ip_{i}\right)+p_{j}=m+p_{j}.$$

Let k denote the greatest integer such that 1<k≤m and $p_{k}>0$. Then, $p_{i}=0$ for k+1≤i≤j−1, and $q_{i}=0$ for k+1≤i≤j. Thus,

$$\sum_{i=k+1}^{j-1}|i-m|p_{i}=\sum_{i=k+1}^{j}\left|i-m^{\prime }\right|q_{i}=0.$$

Also, $0<p_{j}<1$, k≤m and m<j yield $k\leq m<m^{\prime }<m+1<j+1$.

$$\begin{array}{l} AD\left(q\right)=\left(\sum_{i=1}^{k}(m^{\prime }-i)p_{i}\right)+\left(\sum_{i=k+1}^{j}|i-m^{\prime }|q_{i}\right)+\left(\left(j+1\right)-m^{\prime }\;\right)\left(p_{j}+p_{j+1}\right)+\left(\sum_{i=j+2}^{n}(i-m^{\prime })p_{i}\right)\\ =\left(\sum_{i=1}^{k}(m^{\prime }-i)p_{i}\right)+\left(\sum_{i=k+1}^{j-1}|i-m|p_{i}\right)+\left(\left(j+1\right)-m^{\prime }\;\right)\left(p_{j}+p_{j+1}\right)+\left(\sum_{i=j+2}^{n}(i-m^{\prime })p_{i}\right)\\ =\left(\left(\sum_{i=1}^{k}(m-i)p_{i}\right)+p_{j}\left(\sum_{i=1}^{k}p_{i}\right)\right)+\left(\sum_{i=k+1}^{j-1\;}|i-m|p_{i}\right)+\left(\left(j+1\right)-m\right)\left(p_{j}+p_{j+1}\right)-p_{j}\left(p_{j}+p_{j+1}\right)+\left(\left(\sum_{i=j+2}^{n}(i-m)p_{i}\right)-p_{j}\left(\sum_{i=j+2}^{n}p_{i}\right)\right)\\ =\left(\sum_{i=1}^{k}(m-i)p_{i}\right)+p_{j}\left(\sum_{i=1}^{k}p_{i}\right)+\left(\sum_{i=k+1}^{j-1\;}|i-m|p_{i}\right)+\left(j-m\right)p_{j}+p_{j}+\left(\left(j+1\right)-m\right)p_{j+1}-p_{j}\left(p_{j}+p_{j+1}\right)+\left(\sum_{i=j+2}^{n}(i-m)p_{i}\right)-p_{j}\left(\sum_{i=j+2}^{n}p_{i}\right)\\ =AD\left(p\right)+p_{j}\left(\left(\sum_{i=1}^{k}p_{i}\right)+1-\left(p_{j}+p_{j+1}\right)-\left(\sum_{i=j+2}^{n}p_{i}\right)\right)\\ =AD\left(p\right)+p_{j}\left(\left(\sum_{i=1}^{k}p_{i}\right)+1-\sum_{i=j}^{n}p_{i}\right)=AD\left(p\right)+2p_{j}\sum_{i=1}^{k}p_{i}>AD\left(p\right). \end{array}$$

 ☐

Lemmas 3 and 4 were used to derive the upper bound of AD in Corollary 1.

**Lemma** **3.** *Given a distribution*p(x)*over*X={1,2,...,n}*, there exists a distribution*q(x)*with*$q_{1}+q_{n}=1$*and*$q_{i}=0$*for each*i∈X\{1,n}, *satisfying*AD(q)≥AD(p).

**Proof.** First, consider the trivial case of $p_{i}=1$ for some i∈X. Let $q_{1}=1$, then AD(q)=AD(p) holds, obviously. Next, consider the case of $p_{i}<1$ for each i∈X. Let $m=\sum_{i=1}^{n}ip_{i}$ denote the mean of p(x), k denote the greatest integer satisfying 1<k≤m and $p_{k}>0$, and j denote the smallest integer satisfying m<j<n and $p_{j}>0$. We can generate a new distribution q(x) by repeatedly applying Lemma 1 to move each $p_{i\leq k}$ gradually toward $p_{1}$, and by repeatedly applying Lemma 2 to move each $p_{i\geq j}$ gradually toward $p_{n}$. As a result, $q_{1}=\sum_{i=1}^{k}p_{i}$, $q_{n}=\sum_{i=j}^{n}p_{i}$, and $q_{i}=0$ for each i∈X\{1,n}, and AD(q)>AD(p). ☐

**Lemma** **4.** *Given a distribution*p(x)*over*X={1,2,...,n}*. where*$p_{1}+p_{n}=1$*and*$p_{i}=0$*for each*i∈X\{1,n}*,*AD(p)*is maximized when*$p_{1}=p_{n}=0.5$.

**Proof.** Without loss of generality, let $p_{1}=\frac{1}{2}+\delta$ and $p_{n}=\frac{1}{2}-\delta$. for some δ≥0. Then, Equation (3) yields $m=1p_{1}+np_{n}=\left(\frac{1}{2}+\delta \right)+n\left(\frac{1}{2}-\delta \right)=\frac{1+n}{2}+\delta \left(1-n\right)$. If *δ* = 0, then $p_{1}=p_{n}=0.5$. Use $AD_{0}$ to denote the value of AD(p) at *δ* = 0. Then,

$$\begin{array}{l} {AD}_{0}=p_{1}\left(m-1\right)+p_{n}\left(n-m\right)=\frac{1}{2}\left(\frac{1+n}{2}-1\right)+\frac{1}{2}\left(n-\frac{1+n}{2}\right)=\frac{n-1}{2}.\\ AD\left(p\right)=\left(\frac{1}{2}+\delta \right)\left(m-1\right)+\left(\frac{1}{2}-\delta \right)\left(n-m\right)\\ \;=\left(\frac{1}{2}+\delta \right)\left(\frac{1+n}{2}+\delta \left(1-n\right)-1\right)+\left(\frac{1}{2}-\delta \right)\left(n-\frac{1+n}{2}-\delta \left(1-n\right)\right)\\ =\left(\frac{1}{2}+\delta \right)\left(\frac{n-1}{2}+\delta \left(1-n\right)\right)+\left(\frac{1}{2}-\delta \right)\left(\frac{n-1}{2}-\delta \left(1-n\right)\right)\\ =\frac{1}{2}\left(\frac{n-1}{2}+\delta \left(1-n\right)\right)+\delta \left(\frac{n-1}{2}+\delta \left(1-n\right)\right)+\frac{1}{2}\left(\frac{n-1}{2}-\delta \left(1-n\right)\right)-\delta \left(\frac{n-1}{2}-\delta \left(1-n\right)\right)\\ =\frac{n-1}{2}-2\delta^{2}\left(n-1\right)\leq AD_{0}. \end{array}$$

 ☐

**Corollary** **1.**
*Given a probability distribution* p(x) *over* X={1,2,...,n} *,* $0\leq AD\left(p\right)\leq \frac{n-1}{2}$ *holds.*

**Proof.** The upper bound $\frac{n-1}{2}$ is the direct result from Lemmas 3 and 4, and occurs when $p_{1}=p_{n}=0.5$. The lower bound 0 is by the definition of AD(p) in Equation (5), and occurs when $p_{i}=1$ for some i∈X. ☐

## Appendix B

In this section, we derived the range of V(p), where p is a probability distribution over X={1,2,...,n} with mean m. The proof follows similar steps to those in Appendix A.

**Lemma** **5.** *Let*p(x)*and*q(x)*be two probability distributions over*X={1,2,...,n}*,*$p_{i}$*and*$q_{i}$*denote*p(x=i)*and*q(x=i)*, respectively, and*$p_{i}<1$*and*$q_{i}<1$*for each*i∈X*. Let*$m=\sum_{i=1}^{n}ip_{i}$*, and*k*denote the greatest integer satisfying*1<k≤m*and*$p_{k}>0$*. If*$q_{k-1}=p_{k}+p_{k-1}$*,*$q_{k}=0$*, and*$q_{i}=p_{i}$*for each*i∈X\{k−1,k}*, then*V(q)>V(p).

**Proof.** The mean of q(x) is $m^{\prime }=m-p_{k}$. Let j denote the smallest integer such that m<j and $p_{j}>0$. Then, $p_{i}=0$ for k+1≤i≤j−1, and $q_{i}=0$ for k≤i≤j−1. Thus,

$$\sum_{i=k+1}^{j-1}{\left(i-m\right)}^{2}p_{i}=\sum_{i=k}^{j-1}{\left(i-m^{\prime }\right)}^{2}q_{i}=0.$$

Also, $0<p_{k}<1$, k≤m and m<j yield $k-1\leq m-1<m^{\prime }<m<j$.

$$\begin{array}{l} V\left(q\right)=\left(\sum_{i=1}^{k-2}{\left(i-m^{\prime }\right)}^{2}p_{i}\right)+{\left(\left(k-1\right)-m^{\prime }\right)}^{2}\left(p_{k-1}+p_{k}\right)+\left(\sum_{i=k}^{j-1}{\left(i-m^{\prime }\right)}^{2}q_{i}\right)+\left(\sum_{i=j}^{n}{\left(i-m^{\prime }\right)}^{2}p_{i}\right)\\ =\left(\left(\sum_{i=1}^{k-2}{\left(i-m\right)}^{2}p_{i}\right)+2p_{k}\left(\sum_{i=1}^{k-2}\left(i-m\right)p_{i}\right)+p_{k}^{2}\left(\sum_{i=1}^{k-2}p_{i}\right)\right)\\ \;+{\left(\left(k-1\right)-m+p_{k}\right)}^{2}\left(p_{k-1}+p_{k}\right)+(\sum_{i=k+1}^{j-1}{\left(i-m\right)}^{2}p_{i})\\ \;+\left(\left(\sum_{i=j}^{n}{\left(i-m\right)}^{2}p_{i}\right)+2p_{k}\left(\sum_{i=j}^{n}\left(i-m\right)p_{i}\right)+p_{k}^{2}\left(\sum_{i=j}^{n}p_{i}\right)\right)\\ =\left(\sum_{i=1}^{k-2}{\left(i-m\right)}^{2}p_{i}\right)+2p_{k}\left(\sum_{i=1}^{k-2}\left(i-m\right)p_{i}\right)+p_{k}^{2}\left(\sum_{i=1}^{k-2}p_{i}\right)+{\left(\left(k-1\right)-m+p_{k}\right)}^{2}p_{k-1}\\ \;+{\left(k-m+p_{k}-1\right)}^{2}p_{k}+\left(\sum_{i=k+1}^{j-1}{\left(i-m\right)}^{2}p_{i}\right)+\left(\sum_{i=j}^{n}{\left(i-m\right)}^{2}p_{i}\right)+2p_{k}\left(\sum_{i=j}^{n}\left(i-m\right)p_{i}\right)+p_{k}^{2}\left(\sum_{i=j}^{n}p_{i}\right)\\ =\left(\sum_{i=1}^{k-2}{\left(i-m\right)}^{2}p_{i}\right)+2p_{k}\left(\sum_{i=1}^{k-2}\left(i-m\right)p_{i}\right)+p_{k}^{2}\left(\sum_{i=1}^{k-2}p_{i}\right)\\ \;+\left({\left(\left(k-1\right)-m\right)}^{2}+2p_{k}\left(\left(k-1\right)-m\right)+p_{k}^{2}\right)p_{k-1}\\ \;+\left({\left(k-m\right)}^{2}+2\left(k-m\right)\left(p_{k}-1\right)+{\left(p_{k}-1\right)}^{2}\right)p_{k}+\left(\sum_{i=k+1}^{j-1}{\left(i-m\right)}^{2}p_{i}\right)\\ \;+\left(\sum_{i=j}^{n}{\left(i-m\right)}^{2}p_{i}\right)+2p_{k}\left(\sum_{i=j}^{n}\left(i-m\right)p_{i}\right)+p_{k}^{2}\left(\sum_{i=j}^{n}p_{i}\right)\\ =V\left(p\right)+2p_{k}(\left(\sum_{i=1}^{k-2}\left(i-m\right)p_{i}\right)+\left(\left(k-1\right)-m\right)p_{k-1}+\left(k-m\right)\left(p_{k}-1\right)-p_{k}\\ \;+\left(\sum_{i=j}^{n}\left(i-m\right)p_{i}\right))+p_{k}^{2}\left(\left(\sum_{i=1}^{k-2}p_{i}\right)+p_{k-1}+p_{k}+\left(\sum_{i=j}^{n}p_{i}\right)\right)+p_{k}\\ =V\left(p\right)+2p_{k}\left(\left(\sum_{i=1}^{n}\left(i-m\right)p_{i}\right)-p_{k}\right)+p_{k}^{2}\left(\sum_{i=1}^{n}p_{i}\right)+p_{k}\\ =V\left(p\right)-2p_{k}^{2}+p_{k}^{2}+p_{k}=V\left(p\right)+p_{k}\left(1-p_{k}\right)>V\left(p\right). \end{array}$$

 ☐

**Lemma** **6.** *Let*p(x)*and*q(x)*be two probability distributions over*X={1,2,...,n}*,*$p_{i}$*and*$q_{i}$*denote*p(x=i)*and*q(x=i)*, respectively, and*$p_{i}<1$*and*$q_{i}<1$*for each*i∈X*. Let*$m=\sum_{i=1}^{n}ip_{i}$*, and*j*denote the smallest integer satisfying*m<j<n*and*$p_{j}>0$*. If*$q_{j}=0$*,*$q_{j+1}=p_{j}+p_{j+1}$*, and*$q_{i}=p_{i}$*for each*i∈X\{j,j+1}*, then*V(q)>V(p).

**Proof.** The mean of q(x) is $m^{\prime }=m+p_{j}$. Let k denote the greatest integer such that 1<k≤m and $p_{k}>0$. Then, $p_{i}=0$ for k+1≤i≤j−1, and $q_{i}=0$ for k+1≤i≤j. Thus,

$$\sum_{i=k+1}^{j-1}{\left(i-m\right)}^{2}p_{i}=\sum_{i=k+1}^{j}{\left(i-m^{\prime }\right)}^{2}q_{i}=0.$$

Also, $0<p_{j}<1$, k≤m and m<j yield $k\leq m<m^{\prime }<m+1<j+1$.

$$\begin{array}{l} V\left(q\right)=\left(\sum_{i=1}^{k}{\left(i-m^{\prime }\right)}^{2}p_{i}\right)+\left(\sum_{i=k+1}^{j}{\left(i-m^{\prime }\right)}^{2}q_{i}\right)+{\left(\left(j+1\right)-m^{\prime }\right)}^{2}\left(p_{j}+p_{j+1}\right)\\ \;+\left(\sum_{i=j+2}^{n}{\left(i-m^{\prime }\right)}^{2}p_{i}\right)\\ =\left(\left(\sum_{i=1}^{k}{\left(i-m\right)}^{2}p_{i}\right)-2p_{j}\left(\sum_{i=1}^{k}\left(i-m\right)p_{i}\right)+p_{j}^{2}\left(\sum_{i=1}^{k}p_{i}\right)\right)+\sum_{i=k+1}^{j-1}{\left(i-m\right)}^{2}p_{i}\\ \;+{\left(\left(j+1\right)-m-p_{j}\right)}^{2}\left(p_{j}+p_{j+1}\right)\\ \;+\left(\left(\sum_{i=j+2}^{n}{\left(i-m\right)}^{2}p_{i}\right)-2p_{j}\left(\sum_{i=j+2}^{n}\left(i-m\right)p_{i}\right)+p_{j}^{2}\left(\sum_{i=j+2}^{n}p_{i}\right)\right)\\ =\left(\sum_{i=1}^{k}{\left(i-m\right)}^{2}p_{i}\right)-2p_{j}\left(\sum_{i=1}^{k}\left(i-m\right)p_{i}\right)+p_{j}^{2}\left(\sum_{i=1}^{k}p_{i}\right)+\sum_{i=k+1}^{j-1}{\left(i-m\right)}^{2}p_{i}\\ \;+{\left(\left(j-m\right)+\left(1-p_{j}\right)\right)}^{2}p_{j}+{\left(\left(j+1-m\right)-p_{j}\right)}^{2}p_{j+1}+\left(\sum_{i=j+2}^{n}{\left(i-m\right)}^{2}p_{i}\right)\\ \;-2p_{j}\left(\sum_{i=j+2}^{n}\left(i-m\right)p_{i}\right)+p_{j}^{2}\left(\sum_{i=j+2}^{n}p_{i}\right)\\ =\left(\sum_{i=1}^{k}{\left(i-m\right)}^{2}p_{i}\right)-2p_{j}\left(\sum_{i=1}^{k}\left(i-m\right)p_{i}\right)+p_{j}^{2}\left(\sum_{i=1}^{k}p_{i}\right)+\sum_{i=k+1}^{j-1}{\left(i-m\right)}^{2}p_{i}\\ \;+({\left(j-m\right)}^{2}+2\left(j-m\right)\left(1-p_{j}\right)+{\left(1-p_{j}\right)}^{2})p_{j}\\ \;+\left({\left(j+1-m\right)}^{2}-2p_{j}\left(j+1-m\right)+p_{j}^{2}\right)p_{j+1}+(\sum_{i=j+2}^{n}{\left(i-m\right)}^{2}p_{i})\\ \;-2p_{j}\left(\sum_{i=j+2}^{n}\left(i-m\right)p_{i}\right)+p_{j}^{2}\left(\sum_{i=j+2}^{n}p_{i}\right)\\ =V\left(p\right)-2p_{j}\left(\sum_{i=1}^{k}\left(i-m\right)p_{i}\right)+p_{j}^{2}\left(\sum_{i=1}^{k}p_{i}\right)-2p_{j}\left(p_{j}\left(j-m\right)+p_{j+1}\left(j+1-m\right)\right)\\ \;+p_{j}^{2}\left(p_{j}+p_{j+1}\right)+p_{j}\left(2j-2m+1-2p_{j}\right)-2p_{j}\left(\sum_{i=j+2}^{n}\left(i-m\right)p_{i}\right)\\ \;+p_{j}^{2}\left(\sum_{i=j+2}^{n}p_{i}\right)\\ =V\left(p\right)-2p_{j}\left(\sum_{i=1}^{n}\left(i-m\right)p_{i}\right)+p_{j}^{2}\left(\sum_{i=1}^{n}p_{i}\right)+p_{j}\left(2j-2m+1-2p_{j}\right)\\ =V\left(p\right)+p_{j}^{2}+2jp_{j}-2mp_{j}+p_{j}-2p_{j}^{2}\\ =V\left(p\right)+2p_{j}\left(j-m\right)+p_{j}\left(1-p_{j}\right)>V\left(p\right). \end{array}$$

 ☐

**Lemma** **7.** *Given a distribution*p(x)*over*X={1,2,...,n}*, there exists a distribution*q(x)*with*$q_{1}+q_{n}=1$*and*$q_{i}=0$*for each*i∈X\{1,n}, *satisfying*V(q)≥V(p).

**Proof.** First, consider the trivial case of $p_{i}=1$ for some i∈X. Let $q_{1}=1$, then V(q)=V(p) holds, obviously. Next, consider the case of $p_{i}<1$ for each i∈X. Let $m=\sum_{i=1}^{n}ip_{i}$ denote the mean of p(x), k denote the greatest integer satisfying 1<k≤m and $p_{k}>0$, and j denote the smallest integer satisfying m<j<n and $p_{j}>0$. We can generate a new distribution q(x) by repeatedly applying Lemma 5 to move each $p_{i\leq k}$ gradually toward $p_{1}$, and by repeatedly applying Lemma 6 to move each $p_{i\geq j}$ gradually toward $p_{n}$. As a result, $q_{1}=\sum_{i=1}^{k\;}p_{i}$, $q_{n}=\sum_{i=j}^{n}p_{i}$, and $q_{i}=0$ for each i∈X\{1,n}, and V(q)>V(p). ☐

**Lemma** **8.** *Given a distribution*p(x)*over*X={1,2,...,n}*where*$p_{1}+p_{n}=1$*and*$p_{i}=0$*for each*i∈X\{1,n}*,*V(p)*is maximized when*$p_{1}=p_{n}=0.5$.

**Proof.** Without loss of generality, let $p_{1}=\frac{1}{2}+\delta$ and $p_{n}=\frac{1}{2}-\delta$ for some δ≥0. Then, Equation (3) yields $m=1p_{1}+np_{n}=\left(\frac{1}{2}+\delta \right)+n\left(\frac{1}{2}-\delta \right)=\frac{1+n}{2}+\delta \left(1-n\right)$. If δ=0, then $p_{1}=p_{n}=0.5$. Use $V_{0}$ to denote the value of V(p) at δ=0. Then,

$$\begin{array}{l} V_{0}=p_{1}{\left(1-m\right)}^{2}+p_{n}{\left(n-m\right)}^{2}=\frac{1}{2}{\left(\frac{1+n}{2}-1\right)}^{2}+\frac{1}{2}{\left(n-\frac{1+n}{2}\right)}^{2}={\left(\frac{n-1}{2}\right)}^{2}.\\ V\left(p\right)=\left(\frac{1}{2}+\delta \right){\left(m-1\right)}^{2}+\left(\frac{1}{2}-\delta \right){\left(n-m\right)}^{2}\\ \;=\left(\frac{1}{2}+\delta \right){\left(\frac{1+n}{2}+\delta \left(1-n\right)-1\right)}^{2}+\left(\frac{1}{2}-\delta \right){\left(n-\frac{1+n}{2}-\delta \left(1-n\right)\right)}^{2}\\ =\left(\frac{1}{2}+\delta \right){\left(\frac{n-1}{2}+\delta \left(1-n\right)\right)}^{2}+\left(\frac{1}{2}-\delta \right){\left(\frac{n-1}{2}-\delta \left(1-n\right)\right)}^{2}\\ =\frac{1}{2}{\left(\frac{n-1}{2}+\delta \left(1-n\right)\right)}^{2}+\delta {\left(\frac{n-1}{2}+\delta \left(1-n\right)\right)}^{2}+\frac{1}{2}{\left(\frac{n-1}{2}-\delta \left(1-n\right)\right)}^{2}\\ \;-\delta {\left(\frac{n-1}{2}-\delta \left(1-n\right)\right)}^{2}\\ ={\left(\frac{n-1}{2}\right)}^{2}+\delta^{2}{\left(1-n\right)}^{2}+4\delta^{2}\left(1-n\right)\left(\frac{n-1}{2}\right)\\ ={\left(\frac{n-1}{2}\right)}^{2}-\delta^{2}{\left(1-n\right)}^{2}\leq V_{0}. \end{array}$$

 ☐

**Corollary** **2.**
*Given a probability distribution* p(x) *over* X={1,2,...,n} *,* $0\leq V\left(p\right)\leq {\left(\frac{n-1}{2}\right)}^{2}$ *holds.*

**Proof.** The upper bound ${\left(\frac{n-1}{2}\right)}^{2}$ is the direct result from Lemmas 7 and 8, and occurs when $p_{1}=p_{n}=0.5$. The lower bound 0 is by the definition of V(p) in Equation (6), and occurs when $p_{i}=1$ for some i∈X. ☐

## Appendix C

In this section, we derived the range of S(p), where p is a probability distribution over X={1,2,...,n} with mean m. First, Lemma 9 is used to split the probability at x=j into the probabilities at x=1 and at x=⎣m⎦ for 1<j<⎣m⎦. We can repeatedly apply Lemma 9 until $p_{j}=0$ for 1<j<⎣m⎦, and yield a new probability distribution q such that S(q)>S(p).

**Lemma** **9.** *Let*p(x)*be a probability distribution over*X={1,2,...,n}*. Let*$m=\sum_{i=1}^{n}ip_{i}$*and*k=⎣m⎦*. If there exists*$p_{j}>0$*where*1<j<k*, then*S(q)>S(p)*where*q(x)*is a probability distribution over*X*with*$q_{1}=p_{1}+\frac{k-j}{k-1}p_{j}$*,*$q_{j}=0$*,*$q_{k}=p_{k}+\frac{j-1}{k-1}p_{j}$*, and*$q_{i}=p_{i}$*for*i∈X\{1,j,k}.

**Proof.** By Equation (3), the mean of q(x) is also m.

$$\begin{array}{l} S\left(q\right)=\left(\sum_{i\neq 1,j,k}{\left|m-i\right|}^{3}p_{i}\right)+{\left(m-1\right)}^{3}\left(p_{1}+\frac{k-j}{k-1}p_{j}\right)+{\left(m-j\right)}^{3}\left(0\right)+{\left(m-k\right)}^{3}\left(p_{k}+\frac{j-1}{k-1}p_{j}\right)\\ =\left(\sum_{i\neq 1,j,k}{\left|m-i\right|}^{3}p_{i}\right)+{\left(m-1\right)}^{3}p_{1}+{\left(m-1\right)}^{3}\left(\frac{k-j}{k-1}p_{j}\right)+{\left(m-k\right)}^{3}p_{k}+{\left(m-k\right)}^{3}\left(\frac{j-1}{k-1}p_{j}\right)\\ =\left(\sum_{i\neq j}{\left|m-i\right|}^{3}p_{i}\right)+{\left(m-1\right)}^{3}\left(\frac{k-j}{k-1}p_{j}\right)+{\left(m-k\right)}^{3}\left(\frac{j-1}{k-1}p_{j}\right)\\ =\left(\sum_{i\neq j}{\left|m-i\right|}^{3}p_{i}\right)+\left(\frac{p_{j}}{k-1}\right)\left({\left(m-1\right)}^{3}\left(k-j\right)+{\left(m-k\right)}^{3}\left(j-1\right)\right)\\ =\left(\sum_{i\neq j}{\left|m-i\right|}^{3}p_{i}\right)\\ \;+\left(\frac{p_{j}}{k-1}\right)\left(\left(m^{3}-3m^{2}+3m-1\right)\left(k-j\right)+\left(m^{3}-3m^{2}k+3mk^{2}-k^{3}\right)\left(j-1\right)\right)\\ =\left(\sum_{i\neq j}{\left|m-i\right|}^{3}p_{i}\right)\\ \;+\left(\frac{p_{j}}{k-1}\right)(m^{3}k-3m^{2}k+3mk-k-m^{3}j+3m^{2}j-3mj+j+m^{3}j-3m^{2}kj\\ \;+3mk^{2}j-k^{3}j-m^{3}+3m^{2}k-3mk^{2}+k^{3})\\ =(\sum_{i\neq j}{\left|m-i\right|}^{3}p_{i})\\ \;+\left(\frac{p_{j}}{k-1}\right)(\left(m^{3}k-m^{3}\right)+\left(3mk-3mk^{2}\right)+\left(k^{3}-k\right)+\left(3m^{2}j-3m^{2}kj\right)\\ \;+\left(3mk^{2}j-3mj\right)+\left(j-k^{3}j\right))\\ =(\sum_{i\neq j}{\left|m-i\right|}^{3}p_{i})\\ \;+\left(\frac{p_{j}}{k-1}\right)(m^{3}\left(k-1\right)+3mk\left(1-k\right)+k\left(k^{2}-1\right)+3m^{2}j\left(1-k\right)+3mj\left(k^{2}-1\right)\\ \;+j\left(1-k^{3}\right))\\ =(\sum_{i\neq j}{\left|m-i\right|}^{3}p_{i})+p_{j}(m^{3}-3mk+k\left(k+1\right)-3m^{2}j+3mj\left(k+1\right)-j\left(k^{2}+k+1\right))\\ =(\sum_{i\neq j}{\left|m-i\right|}^{3}p_{i})\\ \;+p_{j}(\left(m^{3}-3m^{2}j+3mj^{2}-j^{3}\right)-3mj^{2}+j^{3}-3mk+k\left(k+1\right)+3mj\left(k+1\right)\\ \;-j\left(k^{2}+k+1\right))\\ =S\left(p\right)+p_{j}\left(-3mj^{2}+j^{3}-3mk+k\left(k+1\right)+3mj\left(k+1\right)-j\left(k^{2}+k+1\right)\right)\\ =S\left(p\right)+p_{j}\left(3m\left(-j^{2}-k+j\left(k+1\right)\right)+k\left(k+1\right)\left(1-j\right)+j\left(j^{2}-1\right)\right)\\ =S\left(p\right)+p_{j}\left(3m\left(j-1\right)\left(k-j\right)-k\left(k+1\right)\left(j-1\right)+j\left(j+1\right)\left(j-1\right)\right)\\ =S\left(p\right)+p_{j}\left(j-1\right)\left(3m\left(k-j\right)-k\left(k+1\right)+j\left(j+1\right)\right)\\ =S\left(p\right)+p_{j}\left(j-1\right)\left(3m\left(k-j\right)-\left(k^{2}-j^{2}\right)-\left(k-j\right)\right)\\ =S\left(p\right)+p_{j}\left(j-1\right)\left(k-j\right)\left(3m-\left(k+j\right)-1\right). \end{array}$$

Then 1<j<k=m≤m<n yields 3m−(k+j)−1>0. Thus, S(q)>S(p). ☐

Similar to Lemma 9, Lemma 10 is used to split the probability at x=j into the probabilities at x=⎡m⎤ and at x=n for ⎡m⎤<j<n. We can repeatedly apply Lemma 10 until $p_{j}=0$ for ⎡m⎤<j<n, and yield a new probability distribution q such that S(q)>S(p).

**Lemma** **10.** *Let*p(x)*be a probability distribution over*X={1,2,...,n}*. Let*$m=\sum_{i=1}^{n}ip_{i}$*and*k=m*. If there exists*$p_{j}>0$*where*k<j<n*, then*S(q)>S(p)*where*q(x)*is a probability distribution over*X*with*$q_{k}=p_{k}+\frac{n-j}{n-k}p_{j}$*,*$q_{j}=0$*,*$q_{n}=p_{n}+\frac{j-k}{n-k}p_{j}$*, and*$q_{i}=p_{i}$*for*i∈X\{k,j, n}.

**Proof.** By Equation (3), the mean of q(x) is also m.

$$\begin{array}{l} S\left(q\right)=(\sum_{i\neq k,j,\;n}{\left|m-i\right|}^{3}p_{i})+{\left(k-m\right)}^{3}\left(p_{k}+\frac{n-j}{n-k}p_{j}\right)+{\left(j-m\right)}^{3}\left(0\right)\\ \;+{\left(n-m\right)}^{3}\left(p_{n}+\frac{j-k}{n-k}p_{j}\right)\\ =\left(\sum_{i\neq k,j,\;n}{\left|m-i\right|}^{3}p_{i}\right)+{\left(k-m\right)}^{3}p_{k}+{\left(k-m\right)}^{3}\left(\frac{n-j}{n-k}p_{j}\right)+{\left(n-m\right)}^{3}p_{n}\\ \;+{\left(n-m\right)}^{3}\left(\frac{j-k}{n-k}p_{j}\right)\\ =\left(\sum_{i\neq j}{\left|m-i\right|}^{3}p_{i}\right)+{\left(k-m\right)}^{3}\left(\frac{n-j}{n-k}p_{j}\right)+{\left(n-m\right)}^{3}\left(\frac{j-k}{n-k}p_{j}\right)\\ =\left(\sum_{i\neq j}{\left|m-i\right|}^{3}p_{i}\right)+\left(\frac{p_{j}}{n-k}\right)\left({\left(k-m\right)}^{3}\left(n-j\right)+{\left(n-m\right)}^{3}\left(j-k\right)\right)\\ =\left(\sum_{i\neq j}{\left|m-i\right|}^{3}p_{i}\right)\\ \;+\left(\frac{p_{j}}{n-k}\right)(\left(k^{3}-3k^{2}m+3km^{2}-m^{3}\right)\left(n-j\right)\\ \;+\left(n^{3}-3n^{2}m+3nm^{2}-m^{3}\right)\left(j-k\right))\\ =\left(\sum_{i\neq j}{\left|m-i\right|}^{3}p_{i}\right)\\ \;+\left(\frac{p_{j}}{n-k}\right)(k^{3}n-3k^{2}mn+3km^{2}n-m^{3}n-k^{3}j+3k^{2}mj-3km^{2}j+m^{3}j+n^{3}j\\ \;-3n^{2}mj+3nm^{2}j-m^{3}j-n^{3}k+3n^{2}mk-3nm^{2}k+m^{3}k)\\ =\left(\sum_{i\neq j}{\left|m-i\right|}^{3}p_{i}\right)\\ \;+\left(\frac{p_{j}}{n-k}\right)(\left(-3k^{2}mn+3n^{2}mk\right)+\left(3k^{2}mj-3n^{2}mj\right)+\left(-3km^{2}j+3nm^{2}j\right)\\ \;+\left(-m^{3}n+m^{3}k\right)+\left(-k^{3}j+n^{3}j\right)+\left(k^{3}n-n^{3}k\right))\\ =(\sum_{i\neq j}{\left|m-i\right|}^{3}p_{i})\\ \;+\left(\frac{p_{j}}{n-k}\right)(3kmn\left(n-k\right)-3mj\left(n^{2}-k^{2}\right)+3m^{2}j\left(n-k\right)-m^{3}\left(n-k\right)\\ \;+j\left(n^{3}-k^{3}\right)-nk\left(n^{2}-k^{2}\right))\\ =\left(\sum_{i\neq j}{\left|m-i\right|}^{3}p_{i}\right)+p_{j}\left(3kmn-3mj\left(n+k\right)+3m^{2}j-m^{3}+j\left(n^{2}+nk+k^{2}\right)-nk\left(n+k\right)\right)\\ =(\sum_{i\neq j}{\left|m-i\right|}^{3}p_{i})\\ \;+p_{j}(\left(j^{3}-3mj^{2}+3m^{2}j-m^{3}\right)+3mj^{2}-j^{3}+3kmn-3mj\left(n+k\right)\\ \;+j\left(n^{2}+nk+k^{2}\right)-nk\left(n+k\right))\\ =S\left(p\right)+p_{j}\left(3mj^{2}-j^{3}+3kmn-3mj\left(n+k\right)+j\left(n^{2}+nk+k^{2}\right)-nk\left(n+k\right)\right)\\ =S\left(p\right)+p_{j}\left(3m\left(j-k\right)\left(j-n\right)+n\left(n+k\right)\left(j-k\right)-j\left(j^{2}-k^{2}\right)\right)\\ =S\left(p\right)+p_{j}\left(j-k\right)\left(3m\left(j-n\right)+n\left(n+k\right)-j\left(j+k\right)\right)\\ =S\left(p\right)+p_{j}\left(j-k\right)\left(3m\left(j-n\right)+\left(n^{2}-j^{2}\right)+k\left(n-j\right)\right)\\ =S\left(p\right)+p_{j}\left(j-k\right)\left(n-j\right)\left(-3m+\left(n+j\right)+k\right). \end{array}$$

Then 1<m≤⎡m⎤=k<j<n yields −3m+(n+j)+k>0. Thus, S(q)>S(p). ☐

Lemmas 11, 12, and 13 are used to split the probabilities at x=m, x=⎣m⎦, and x=⎡m⎤, respectively, into x=1 and x=n.

**Lemma** **11.** *Let*p(x)*be a probability distribution over*X={1,2,...,n}*. Let*$m=\sum_{i=1}^{n}ip_{i}$*. If*m∈X*and*$p_{m}>0$*, then*S(q)>S(p)*where*q(x)*is a probability distribution over*X*with*$q_{1}=p_{1}+\frac{n-m}{n-1}p_{m}$*,*$q_{m}=0$*,*$q_{n}=p_{n}+\frac{m-1}{n-1}p_{m}$*, and*$q_{i}=p_{i}$*for*i∈X\{1,m, n}.

**Proof.** By Equation (3), the mean of q(x) is also m.

$$\begin{array}{l} S\left(q\right)=(\sum_{i\neq 1,m,\;n}{\left|m-i\right|}^{3}p_{i})+{\left(m-1\right)}^{3}\left(p_{1}+\frac{n-m}{n-1}p_{m}\right)+{\left|m-m\right|}^{3}(0)\\ \;+{\left(n-m\right)}^{3}\left(p_{n}+\frac{m-1}{n-1}p_{m}\right)\\ =S\left(p\right)+{\left(m-1\right)}^{3}\left(\frac{n-m}{n-1}p_{m}\right)+{\left(n-m\right)}^{3}\left(\frac{m-1}{n-1}p_{m}\right)\\ =S\left(p\right)+\frac{p_{m}\left(m-1\right)\left(n-m\right)}{n-1}\left({\left(m-1\right)}^{2}+{\left(n-m\right)}^{2}\right)\;>S\left(p\right). \end{array}$$

 ☐

**Lemma** **12.** *Let*p(x)*be a probability distribution over*X={1,2,...,n}*,*$m=\sum_{i=1}^{n}ip_{i}$*, and*k=⎣m⎦*. If*1<k<m*and*$p_{k}>0$*, then*S(q)>S(p)*where*q(x)*is a probability distribution over*X*with*$q_{1}=p_{1}+\frac{n-k}{n-1}p_{k}$*,*$q_{k}=0$*,*$q_{n}=p_{n}+\frac{k-1}{n-1}p_{k}$*, and*$q_{i}=p_{i}$*for*i∈X\{1,k,n}.

**Proof.** By Equation (3), the mean of q(x) is also m.

$$\begin{array}{l} S\left(q\right)=\left(\sum_{i\neq 1,k,n}{\left|m-i\right|}^{3}p_{i}\right)+{\left(m-1\right)}^{3}\left(p_{1}+\frac{n-k}{n-1}p_{k}\right)+{\left(m-k\right)}^{3}\left(0\right)+{\left(n-m\right)}^{3}\left(p_{n}+\frac{k-1}{n-1}p_{k}\right)\\ =S\left(p\right)+{\left(m-1\right)}^{3}\left(\frac{n-k}{n-1}p_{k}\right)+{\left(n-m\right)}^{3}\left(\frac{k-1}{n-1}p_{k}\right)-{\left(m-k\right)}^{3}p_{k}\\ =S\left(p\right)+\frac{p_{k}}{n-1}\left({\left(m-1\right)}^{3}\left(n-k\right)+{\left(n-m\right)}^{3}\left(k-1\right)-{\left(m-k\right)}^{3}\left(n-1\right)\right)\\ =S\left(p\right)+\frac{p_{k}}{n-1}\left({\left(m-1\right)}^{3}\left(n-k\right)+{\left(n-m\right)}^{3}\left(k-1\right)-{\left(m-k\right)}^{3}\left(n-k+k-1\right)\right)\\ =S\left(p\right)+\frac{p_{k}}{n-1}\left({\left(m-1\right)}^{3}\left(n-k\right)-{\left(m-k\right)}^{3}\left(n-k\right)+{\left(n-m\right)}^{3}\left(k-1\right)-{\left(m-k\right)}^{3}\left(k-1\right)\right)\\ =S\left(p\right)+\frac{p_{k}}{n-1}\left({\left(m-k+k-1\right)}^{3}\left(n-k\right)-{\left(m-k\right)}^{3}\left(n-k\right)+{\left(n-m\right)}^{3}\left(k-1\right)-{\left(m-k\right)}^{3}\left(k-1\right)\right)\\ >S\left(p\right)+\frac{p_{k}}{n-1}\left({\left(k-1\right)}^{3}\left(n-k\right)+{\left(n-m\right)}^{3}\left(k-1\right)-{\left(m-k\right)}^{3}\left(k-1\right)\right)\\ =S\left(p\right)+\frac{p_{k}\left(k-1\right)}{n-1}\left({\left(k-1\right)}^{2}\left(n-k\right)+{\left(n-m\right)}^{3}-{\left(m-k\right)}^{3}\right). \end{array}$$

Then, k−1≥1>m−k>0 and n>m yield ${\left(k-1\right)}^{2}>{\left(m-k\right)}^{2}>0$ and n−k>m−k, and thus, ${\left(k-1\right)}^{2}\left(n-k\right)>{\left(m-k\right)}^{3}$. Therefore, S(q)>S(p) holds. ☐

**Lemma** **13.** *Let*p(x)*be a probability distribution over*X={1,2,...,n}*,*$m=\sum_{i=1}^{n}ip_{i}$*, and*k=⎡m⎤*. If*m<k<n*and*$p_{k}>0$*, then*S(q)>S(p)*where*q(x)*is a probability distribution over*X*with*$q_{1}=p_{1}+\frac{n-k}{n-1}p_{k}$*,*$q_{k}=0$*,*$q_{n}=p_{n}+\frac{k-1}{n-1}p_{k}$*, and*$q_{i}=p_{i}$*for*i∈X\{1,k,n}.

**Proof.** By Equation (3), the mean of q(x) is also m.

$$\begin{array}{l} S\left(q\right)=\left(\sum_{i\neq 1,k,n}{\left|m-i\right|}^{3}p_{i}\right)+{\left(m-1\right)}^{3}\left(p_{1}+\frac{n-k}{n-1}p_{k}\right)+{\left(k-m\right)}^{3}\left(0\right)+{\left(n-m\right)}^{3}\left(p_{n}+\frac{k-1}{n-1}p_{k}\right)\\ =S\left(p\right)+{\left(m-1\right)}^{3}\left(\frac{n-k}{n-1}p_{k}\right)+{\left(n-m\right)}^{3}\left(\frac{k-1}{n-1}p_{k}\right)-{\left(k-m\right)}^{3}p_{k}\\ =S\left(p\right)+\frac{p_{k}}{n-1}\left({\left(m-1\right)}^{3}\left(n-k\right)+{\left(n-m\right)}^{3}\left(k-1\right)-{\left(k-m\right)}^{3}\left(n-1\right)\right)\\ =S\left(p\right)+\frac{p_{k}}{n-1}\left({\left(m-1\right)}^{3}\left(n-k\right)+{\left(n-m\right)}^{3}\left(k-1\right)-{\left(k-m\right)}^{3}\left(n-k+k-1\right)\right)\\ =S\left(p\right)+\frac{p_{k}}{n-1}\left({\left(m-1\right)}^{3}\left(n-k\right)-{\left(k-m\right)}^{3}\left(n-k\right)+{\left(n-m\right)}^{3}\left(k-1\right)-{\left(k-m\right)}^{3}\left(k-1\right)\right)\\ =S\left(p\right)+\frac{p_{k}}{n-1}\left({\left(m-1\right)}^{3}\left(n-k\right)-{\left(k-m\right)}^{3}\left(n-k\right)+{\left(n-k+k-m\right)}^{3}\left(k-1\right)-{\left(k-m\right)}^{3}\left(k-1\right)\right)\\ >S\left(p\right)+\frac{p_{k}}{n-1}\left({\left(m-1\right)}^{3}\left(n-k\right)-{\left(k-m\right)}^{3}\left(n-k\right)+{\left(n-k\right)}^{3}\left(k-1\right)\right)\\ =S\left(p\right)+\frac{p_{k}\left(n-k\right)}{n-1}\left({\left(m-1\right)}^{3}-{\left(k-m\right)}^{3}+{\left(n-k\right)}^{2}\left(k-1\right)\right). \end{array}$$

Then, n−k≥1>k−m>0 and m>1 yield ${\left(n-k\right)}^{2}>{\left(k-m\right)}^{2}>0$ and k−1>k−m, and thus, ${\left(n-k\right)}^{2}\left(k-1\right)>{\left(k-m\right)}^{3}$. Therefore, S(q)>S(p) holds. ☐

Given a probability distribution p(x) with the probability concentrating at both ends, Lemma 14 shows that S(p) is maximized when the probability is evenly distributed.

**Lemma** **14.** *Given a probability distribution*p(x)*over*X={1,2,...,n}*where*$p_{1}+p_{n}=1$*and*$p_{i}=0$*for each*i∈X\{1,n}*,*S(p)*is maximized when*$p_{1}=p_{n}=0.5$.

**Proof.** Without loss of generality, let $p_{1}=\frac{1}{2}+\delta$ and $p_{n}=\frac{1}{2}-\delta$ where $0\leq \delta \leq \frac{1}{2}$. Then, Equation (3) yields $m=p_{1}+np_{n}=\left(\frac{1}{2}+\delta \right)+n\left(\frac{1}{2}-\delta \right)=\frac{1+n}{2}+\delta \left(1-n\right)$. Use $S_{0}$ to denote the value of S(p) at δ=0. Then,

$$\begin{array}{l} S_{0}=p_{1}{\left(m-1\right)}^{3}+p_{n}{\left(n-m\right)}^{3}=\frac{1}{2}{\left(\frac{1+n}{2}-1\right)}^{3}+\frac{1}{2}{\left(n-\frac{1+n}{2}\right)}^{3}={\left(\frac{n-1}{2}\right)}^{3}.\\ S\left(p\right)=\left(\frac{1}{2}+\delta \right){\left(m-1\right)}^{3}+\left(\frac{1}{2}-\delta \right){\left(n-m\right)}^{3}\\ =\left(\frac{1}{2}+\delta \right){\left(\frac{1+n}{2}+\delta \left(1-n\right)-1\right)}^{3}+\left(\frac{1}{2}-\delta \right){\left(n-\frac{1+n}{2}-\delta \left(1-n\right)\right)}^{3}\\ =\left(\frac{1}{2}+\delta \right){\left(\frac{n-1}{2}+\delta \left(1-n\right)\right)}^{3}+\left(\frac{1}{2}-\delta \right){\left(\frac{n-1}{2}-\delta \left(1-n\right)\right)}^{3}\\ =\frac{1}{2}{\left(\frac{n-1}{2}+\delta \left(1-n\right)\right)}^{3}+\delta {\left(\frac{n-1}{2}+\delta \left(1-n\right)\right)}^{3}+\frac{1}{2}{\left(\frac{n-1}{2}-\delta \left(1-n\right)\right)}^{3}\\ \;-\delta {\left(\frac{n-1}{2}-\delta \left(1-n\right)\right)}^{3}\\ =\frac{1}{2}\left({\left(\frac{n-1}{2}\right)}^{3}+3{\left(\frac{n-1}{2}\right)}^{2}\delta \left(1-n\right)+3\left(\frac{n-1}{2}\right)\delta^{2}{\left(1-n\right)}^{2}+\delta^{3}{\left(1-n\right)}^{3}\right)\\ \;+\frac{1}{2}\left({\left(\frac{n-1}{2}\right)}^{3}-3{\left(\frac{n-1}{2}\right)}^{2}\delta \left(1-n\right)+3\left(\frac{n-1}{2}\right)\delta^{2}{\left(1-n\right)}^{2}-\delta^{3}{\left(1-n\right)}^{3}\right)\\ \;+\;\delta \left({\left(\frac{n-1}{2}\right)}^{3}+3{\left(\frac{n-1}{2}\right)}^{2}\delta \left(1-n\right)+3\left(\frac{n-1}{2}\right)\delta^{2}{\left(1-n\right)}^{2}+\delta^{3}{\left(1-n\right)}^{3}\right)\\ \;-\delta \left({\left(\frac{n-1}{2}\right)}^{3}-3{\left(\frac{n-1}{2}\right)}^{2}\delta \left(1-n\right)+3\left(\frac{n-1}{2}\right)\delta^{2}{\left(1-n\right)}^{2}-\delta^{3}{\left(1-n\right)}^{3}\right)\\ =\left({\left(\frac{n-1}{2}\right)}^{3}+3\left(\frac{n-1}{2}\right)\delta^{2}{\left(1-n\right)}^{2}\right)+2\delta \left(3{\left(\frac{n-1}{2}\right)}^{2}\delta \left(1-n\right)+\delta^{3}{\left(1-n\right)}^{3}\right)\\ ={\left(\frac{n-1}{2}\right)}^{3}-2\delta^{4}{\left(n-1\right)}^{3}\leq S_{0}. \end{array}$$

☐

**Corollary** **3.**
*Given a probability distribution* p(x) *over* X={1,2,...,n} *,* $0\leq S\left(p\right)\leq {\left(\frac{n-1}{2}\right)}^{3}$ *holds.*

**Proof.** The lower bound 0 is by the definition of S(p) in Equation (6), and occurs when $p_{i}=1$ for some i∈X. The upper bound ${\left(\frac{n-1}{2}\right)}^{3}$ is the direct result from Lemmas 9 to 14, and occurs when $p_{1}=p_{n}=0.5$. First, we can repeatedly apply Lemmas 9 and 10 to yield a new distribution q(x) such that S(q)>S(p) and $q_{j}=0$ for 1<j<⎣m⎦ and for ⎡m⎤<j<n. Then, we apply Lemmas 11, 12, and 13 to yield a new probability distribution r such that S(r)>S(q) and $r_{j}=0$ for 1<j<n. Finally, we apply Lemma 14 to show that $S\left(r\right)\leq S_{0}$ where $S_{0}={\left(\frac{n-1}{2}\right)}^{3}$ is the value of S(r) when $r_{1}=r_{n}=0.5$. ☐
